# Supplementary material for: The interactome of intact mitochondria by cross-linking mass spectrometry provides evidence for coexisting respiratory supercomplexes
Source: Mol Cell Proteomics. 2017 Dec 8;17(2):216–32. doi: 10.1074/mcp.RA117.000470 (PMC5795388; doi:10.1074/mcp.RA117.000470)
Supplement: Supplemental Data [file supp_RA117.000470_133922_0_supp_23978_fzffwf.zip › spectra_annotation/mito_DR_spectra_annotation/418-1-3-1-3-1.pdf]

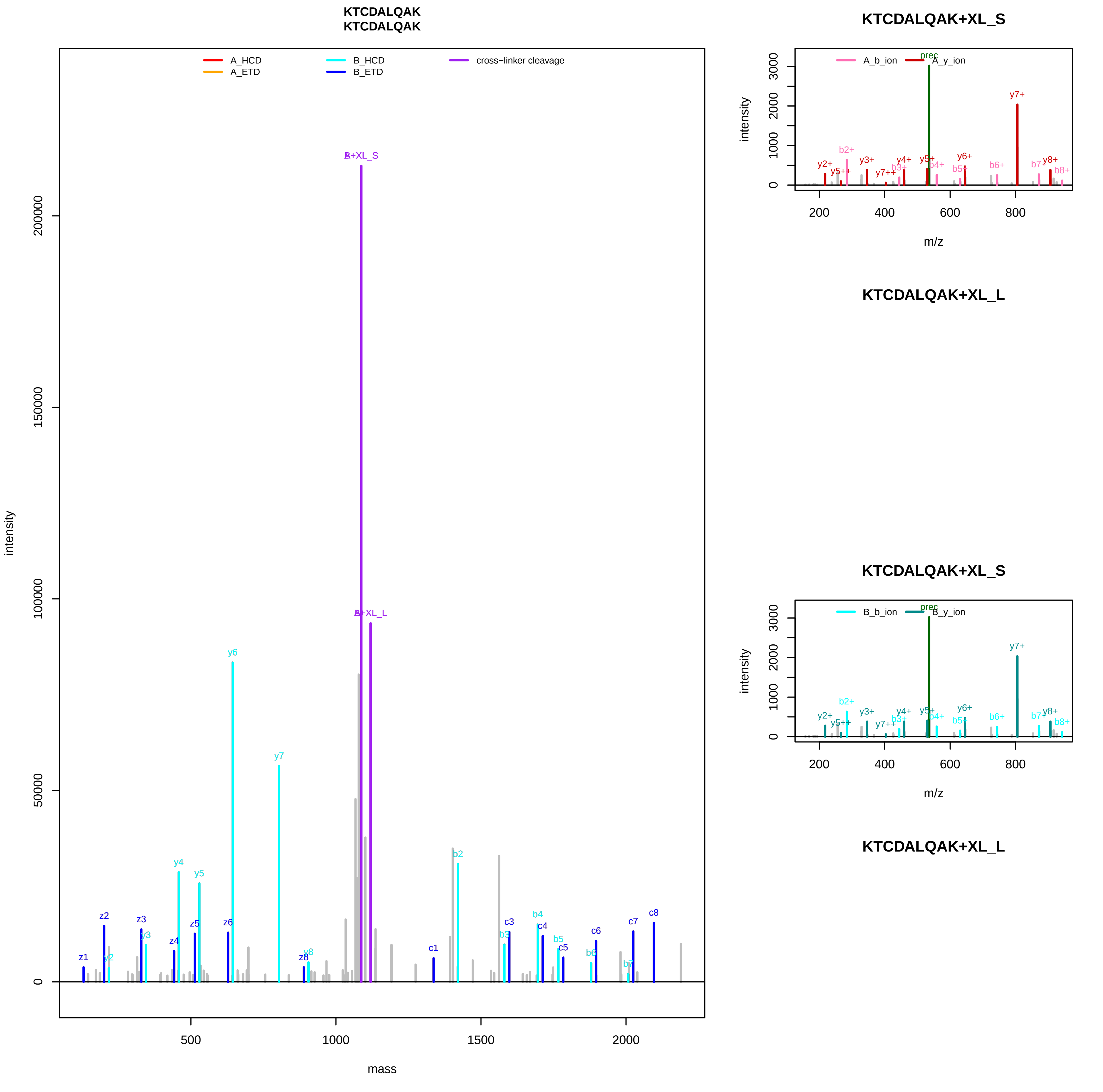
KTCDALQAK
KTCDALQAK
A_HCD
A_ETD
B_HCD
B_ETD
cross−linker cleavage
intensity
mass
0
50000
100000
150000
200000
500
1000
1500
2000
A+XL_S
B+XL_S
A+XL_L
B+XL_L
z1
z2
z3
z4
z5
z6
z8
y2
y3
y4
y5
y6
y7
y8
c1
c3
c4
c5
c6
c7
c8
b2
b3
b4
b5
b6
b7
KTCDALQAK+XL_S
A_b_ion
A_y_ion
prec
intensity
m/z
0
1000
2000
3000
200
400
600
800
y2+
y5++
b2+
y3+
y7++
b3+
y4+
y5+
b4+
b5+
y6+
b6+
y7+
b7+
y8+
b8+
KTCDALQAK+XL_L
KTCDALQAK+XL_S
B_b_ion
B_y_ion
prec
intensity
m/z
0
1000
2000
3000
200
400
600
800
y2+
y5++
b2+
y3+
y7++
b3+
y4+
y5+
b4+
b5+
y6+
b6+
y7+
b7+
y8+
b8+
KTCDALQAK+XL_L
